# Evaluation of the Relationship between Maxillary and Mandibular Third Molar Impaction and Vertical and Anteroposterior Dimensions of the Face 

**DOI:** 10.30476/dentjods.2025.104327.2519

**Published:** 2026-03-01

**Authors:** Mostafa Sheikhi, Mohaddeseh Nasiri, Masoomeh Amani, Leila Abdolhosseinzadeh, Faezeh Zamani

**Affiliations:** 1 Dept. of Orthodontics, School of Dentistry, Zanjan University of Medical Sciences, Zanjan, Iran.; 2 Dept. of Oral and Maxillofacial Surgery, School of Dentistry, Zanjan University of Medical Sciences, Zanjan, Iran.; 3 Dept. of Operative Dentistry, School of Dentistry, Zanjan University of Medical Sciences, Zanjan, Iran.

**Keywords:** Cephalometry, Impacted Tooth, Malocclusion, Third Molar

## Abstract

**Background:**

The impaction of the third molar is associated with specific facial skeletal and dental characteristics. Therefore, determining the type of facial skeletal growth may help predict third molar impaction and assist in treatment planning.

**Purpose:**

This study aimed to investigate the correlation between the impaction of the maxillary and mandibular third molars with the vertical and anteroposterior dimensions of the face, as well as to examine the prevalence and type of impaction.

**Materials and Method:**

In this descriptive-analytical study, panoramic and lateral cephalometric radiographs of 200 patients who were referred to a radiology center in Zanjan were examined. The position, angle, and type of impaction were evaluated on the panoramic radiographs, and the type of vertical and anteroposterior facial profile was determined through lateral cephalometry. Finally, the correlation between impaction and the type of facial profile was analyzed with the Chi-Square test at a significance level of 0.05.

**Results:**

The prevalence of third molar impaction was higher in patients with skeletal Class II and those with a dolichofacial profile (55.5% and 63.5%). Class B, Class II, and mesioangular impactions were more common in the mandible, whereas Class C and distoangular impactions were more prevalent in the maxilla. The level of third molar impaction in the mandible was significantly related to the vertical dimension of the face (*p*= 0.003). The angle of impaction in the mandible was significantly associated with both the vertical and anteroposterior dimensions of the face (*p*= 0.036 and *p*= 0.014).

**Conclusion:**

The findings of this study can be beneficial in orthodontics for evaluating the impact of third molar impaction on dental crowding and facilitating the development of appropriate treatment plans. Knowing which individuals are more susceptible to third molar impaction enables specialists to implement early interventions. Additionally, timely referrals to oral and maxillofacial surgeons can be made when necessary.

## Introduction

Dental impaction occurs when a tooth fails to fully erupt within the expected time frame. Various factors can explain why this happens. Typically, when the eruption path of a tooth is abnormal, it may be obstructed by adjacent teeth, potentially causing damage to these neighboring teeth. A common cause of impaction is a lack of space, which often results in later-erupting teeth becoming impacted [ 1 ].

The third molar, which usually erupts between the ages of 18 and 24, is the most frequently impacted tooth [ 2 - 3 ]. This condition can lead to various issues, including distal caries of the second molar, root resorption of adjacent teeth, pericoronitis, odontogenic cysts, malocclusion and even neoplasms. Moreover, it can adversely affect arch crowding and the stability of orthodontic treatments [ 4 - 7 ]. Given the impact of the third molar on various factors such as anterior tooth crowding and relapse, uprighting or distalization of first and second molars, and anchorage preparation, its consideration in orthodontics is crucial [ 8 ]. Moreover, concerning the impact of the impacted tooth's position on dental treatment planning, complications, adverse effects, treatment costs, and overall oral health improvement, reporting information on this matter seems to be valuable [ 8-9 ].

Contributing factors to impaction include abnormal orientation of adjacent teeth, dense overlying bone, excessive soft tissue, and genetic anomalies, genetic and ethnic factors [ 1 - 2 ]. One major factor in mandibular third molar impaction is insufficient retromolar space (the area between the distal surface of the second molar and the ramus). Other significant factors include the size and rotation of the mandible [ 4 ].

Additionally, facial growth in various spatial dimensions can influence the impaction of permanent teeth [ 3 ]. Some research indicates a relationship between third molar impaction and specific skeletal and dental characteristics. Identifying the type of skeletal growth patterns that may predict third molar impaction could be valuable for treatment planning [ 10 - 11 ]. 

Due to the importance of the relationship between maxillary and mandibular third molar impaction and facial skeletal type in predicting orthodontic treatment outcomes, as well as demographic differences, this study aimed to explore the association between third molar impaction in the maxilla and mandible with vertical and anteroposterior facial dimensions in Zanjan. 

## Materials and Method

This study was a cross-sectional descriptive-analytical investigation. It was conducted on radiographic images of patients who provided informed consent and referred to a private maxillofacial radiology center in Zanjan city. Panoramic and lateral cephalometric radiographs of the patients were collected. The sample size was estimated based on a study by Topkara *et al*. [ 12 ], considering a 95% confidence level and 90% power, based on the prevalence of impacted third molars in the mentioned study (0.541) and a precision level of 0.16. A minimum of 130 records was estimated, but 200 cases were reviewed in our research. The formula used and its various components are as follows:

$N=\frac{z_{1-\frac{\alpha }{2}}^{2}P(1-P)}{d^{2}}={\left(\mathrm{1.96}\right)}^{2}\times \mathrm{0.541}\frac{\left(1-\mathrm{0.541}\right)}{{\left(\mathrm{0.086}\right)}^{2}}=\mathrm{130}$

200 eligible patient records with impaction in either one or both jaws were randomly selected (using a random number table) from the available records and included in the study. The validity and reliability of the radiographs and the various methods of classifying tooth impaction used in previous studies were confirmed. Therefore, a combination of these methods was used in our study [ 13 - 14 ].

Individuals over 18 years of age with suitable and examinable radiographs, where the root of the third molar was obvious, were included in the study. Radiographs of patients lacking demographic information, patients with syndromes (such as Down syndrome and cleidocranial dysplasia) that had distinct radiographic features, or those with a history of facial trauma, were excluded from the study.

To evaluate the position of the mandibular third molar (M3), the following criteria were used:

**Pell and Gregory Classification (Vertical):** This classification divides the depth of impaction of M3 relative to the occlusal plane of the second molar (M2) into three classes:

- Class A: The depth of impaction of M3 is at the level or nearly level of the occlusal plane of M2.
- Class B: The depth of impaction of M3 is between the occlusal plane and the CEJ (Cemento Enamel Junction) of M2.
- Class C: The depth of impaction of M3 is below the CEJ of M2 [ 15 ].

**Pell and Gregory Classification (Horizontal):** This classification assesses the space between the mandibular ramus and M3:

- Class I: There is enough space for the eruption of M3.
- Class II: M3 is partially within the ramus.
- Class III: M3 is entirely within the ramus [ 16 - 17 ].

**Schiller Classification:** This determines the mesial-distal relationship and the angle of impaction. In the mesioangular type, the long axis of the third molar crown forms an angle of 11 to 70 degrees mesially with the occlusal surface of the second molar. If this angle is towards the distal, it is classified as distoangular. An angle less than 10 degrees either mesially or distally is considered vertical, and an angle greater than 70 degrees either mesially or distally is considered horizontal impaction [ 18 - 19 ].

**The modified Archer Classification:** It was used to determine the position of the impaction in the maxilla. Based on the depth of the maxillary third molar (M3) impaction, it is divided into four classes:

- Class A: The lowest part of the third molar crown is aligned with the occlusal plane of the M2.
- Class B: The lowest part of the third molar crown is between the occlusal plane of the M2 and the CEJ.
- Class C: The lowest part of the third molar crown is between the CEJ of the M2 and the middle third of the second molar root.
- Class D: The lowest part of the third molar crown is aligned with or above the apical third of the second molar root [ 20 ].

**Figure 1:**
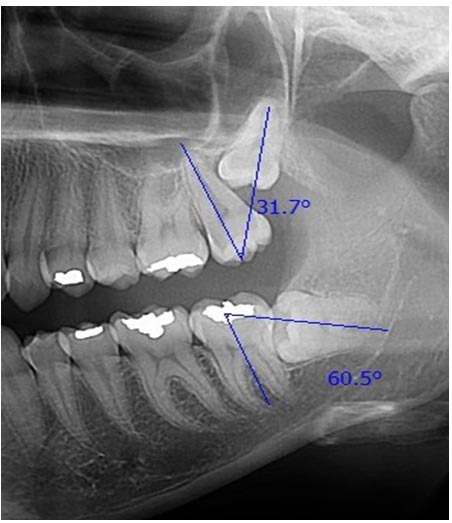
Measurement of impacted third molar angulation

To determine the angle of impaction, the long axis of the maxillary third molar was compared to the long axis of the maxillary second molar, and it was categorized as mesioangular, distoangular, horizontal, or vertical [ 20 ] (Figure 1).

**Figure 2:**
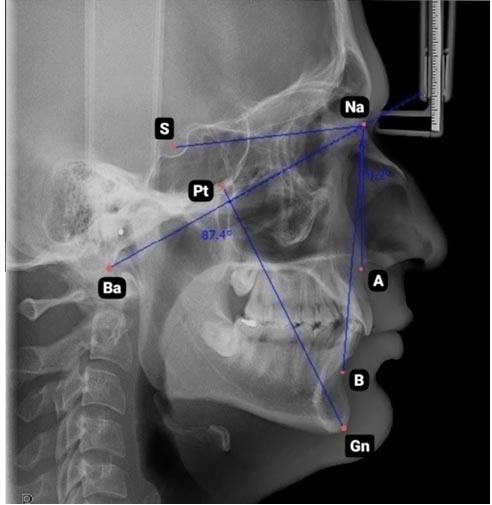
Cephalometric analysis to evaluate facial profile (S: sella; NA: nasion; Pt: pterygomaxillary fissure; Ba: basion; A: a point; B: b point; Gn: gnathion)

The type of vertical facial profile was determined through lateral cephalometric analysis based on the facial axis angle (Ba-Na and Pt-Gn angles) (Figure 2). 

If this angle is greater than 93 degrees, it is classified as brachyfacial; if it is less than 83 degrees, it is classified as dolichofacial; and angles between these two are considered mesofacial [ 13 ].

The anteroposterior facial profile was determined using lateral cephalometry. The sella-nasion-a point angle (SNA), sella-nasion-b point angle (SNB), and a point-nasion-b point angle (ANB) were assessed. A SNA angle greater than 82 degrees indicates a protrusive maxilla, while an angle less than 82 degrees, signifies a retrusive maxilla. A SNB angle less than 80 degrees denotes a retrusive mandible, whereas a SNB angle greater than 80 degrees suggests a prognathic mandible. The normal value of the ANB angle is 2 degrees. Values greater than 2 degrees indicate a tendency toward Class II skeletal relationship. Angles less than 2 degrees and negative values suggest that the mandible is positioned anterior to the maxilla, indicating a Class III skeletal relationship [ 21 ]. In cases where ANB values were unreliable, Wits appraisal was used as an auxiliary method to assess the severity of anteroposterior discrepancies. In normal conditions, the Wits value is -1 mm in males and 0mm in females. The more negative this value, the greater the tendency toward a Class III skeletal relationship, whereas more positive values indicate a tendency toward a Class II skeletal relationship [ 22 ].

Finally, after measuring and recording the data, statistical analysis was performed using SPSS 22 software. Descriptive statistics were reported first, and the Chi-Square test was used to compare variables at a significance level of less than 0.05.

In this study, informed consent was obtained from the patients, and the collected data remained strictly confidential and secured by the researchers. The research was conducted with the approval of the University's Ethics Committee (Ethics Code: IR.ZUMS.REC. 1399.281).

## Results

In this study, 200 selected radiographs, including 500 impacted third molars, were examined. Among these, 351 teeth (70.2%) belonged to female patients and 149 teeth (29.8%) belonged to male patients. The teeth analyzed consisted of 242 (48.4%) impacted maxillary teeth and 258 (51.6%) impacted mandibular teeth.

In the anteroposterior dimension of the facial skeleton, skeletal Class II was the most common in both males and females (63 males (42.3%) and 221 females (63%)), while skeletal Class I was the least common (34 males (22.8%) and 64 females (18.2%)). The most frequent vertical facial type in both genders was dolichofacial (327 teeth (65.4%)), and the least frequent was brachyfacial (48 teeth (9.6%)). 

For mandibular teeth, the most common depth of impaction in both genders was Class B (121 teeth, 46.9%) and Class II (150 teeth, 58.1%). The most frequent angulation of impaction was mesioangular (121 teeth, 46.9%), while distoangular impaction was the least common (28 teeth, 10.9%).

For maxillary teeth, the most common depth of impaction in both genders was Class C (154 teeth, 63.7%), and the most frequent angulation was distoangular (120 teeth, 49.6%).

The results showed no significant association between the depth of impaction and the vertical (*p*= 0.886 for mandible, *p*= 0.182 for maxilla) or anteroposterior (*p*= 0.110 for mandible, *p*= 0.921 for maxilla) dimensions of the facial skeleton (Table 1). Additionally, there was no significant association between the anterior border of the ramus and the anteroposterior dimension (*p*= 0.105), but a significant association was found with the vertical dimension (*p*= 0.003) (Table 2). Furthermore, a significant association was found between the angulation of mandibular third molar impaction and both the vertical (*p*= 0.036) and anteroposterior (*p*= 0.014) dimensions, but no significant association was observed in the maxilla (*p*= 0.120 for vertical, *p*= 0.260 for anteroposterior) (Table 3). 

**Table 1:** Distribution and correlation of impaction depths of M3 according to Pell and Gregory classification (vertical) with vertical and anteroposterior facial dimensions

Parameters		Mandibular M3			*p* Value	Maxillary M3			*p* Value
		A	B	C		B	C	D	
Vertical Facial Type	Brachyfacial	7(29.2%)	11(45.8%)	6(25.0%)	0.886	11(45.8%)	10(41.7%)	3(12.5%)	0.182
	Mesofacial17(25.8%)	31(41.0%)	18(27.3%)	15(25.4%)		39(66.1%)	5(8.5%)		
	Dolichofacial	53(31.5%)	79(47.0%)	36(21.4%)		45(28.3%)	105(66.0%)	9(5.7%)	
Anteroposterior Skeletal Pattern	Cl I	15(30.6%)	21(42.9%)	13(26.5%)	0.110	12(24.5%)	33(67.3%)	4(8.2%)	0.921
	Cl II	40(25.8%)	74(47.7%)	41(26.5%)		40(31.0%)	81(62.8%)	8(6.2%)	
	Cl III	22(40.7%)	26(48.1%)	6(11.1%)		19(29.7%)	40(62.5%)	5(7.8%)	

M3: third molar; Cl: class

**Table 2:** Distribution and Correlation of Impaction Depths of M3 According to Pell and Gregory Classification (Horizontal) with Vertical and Anteroposterior Facial Dimensions

Parameters		Pell and Gregory Classification (Horizontal)			*p* Value
		I	II	III	
Vertical Facial Type	Brachyfacial	8(33.3%)	12(50.0%)	4(16.7%)	0.003
	Mesofacial20(30.3%)	36(54.5%)	10(15.2%)		
	Dolichofacial	62(36.9%)	102(60.7%)	4(2.4%)	
Anteroposterior Skeletal Pattern	Cl I	19(38.8%)	25(51.0%)	5(10.2%)	0.105
	Cl II	46(29.7%)	97(62.6%)	12(7.7%)	
	Cl III	25(46.3%)	28(51.9%)	1(1.9%)	

M3: third molar; Cl: class

**Table 3:** Distribution and Correlation of Impaction Angulation of Mandibular and Maxillary M3 with Vertical and Anteroposterior Facial Dimensions

Parameters		Mandibular M3				*p* Value	Maxillary M3			*p* Value
		Mesioangular	Distoangular	Vertical	Horizontal		Mesioangular	Distoangular	Vertical	
Vertical Facial Type	Brachyfacial	10(41.7%)	0(0%)	3(12.5%)	11(45.8%)	0.036	5(20.8%)	17(70.8%)	2(8.3%)	0.120
Mesofacial	35(53.0%)	5(7.6%)	6(9.1%)	20(30.3%)	13(22.0%)		32(54.2%)	14(23.7%)		
Dolichofacial	76(45.2%)	23(13.7%)	32(19.0%)	37(22.0%)	49(30.8%)		71(44.7%)	39(24.5%)		
Anteroposterior Skeletal Pattern	Cl I	24(49.0%)	7(14.3%)	2(4.1%)	16(32.7%)	0.014	16(32.7%)	19(38.8%)	14(28.6%)	0.260
Cl II	76(49.0%)	16(10.3%)	22(14.2%)	41(26.5%)	35(21.1%)		63(48.8%)	31(24.0%)		
Cl III	21(38.9%)	5(9.3%)	17(31.5%)	11(20.4%)	16(25.0%)		38(59.4%)	10(15.6%)		

M3: third molar; Cl: class

## Discussion

In this study, among the 200 patients examined, the prevalence of third molar impaction in females was more than twice as high as in males. Our study was consistent with the findings of GUL *et al*. [ 4 ] and Abdelaziz *et al*. [ 8 ]. Contributing factors to the higher prevalence in females include differences in jaw growth and smaller jaw dimensions compared to males. In females, jaw growth typically ceases when the third molar begins to erupt, while in males, jaw growth continues, providing more space for the third molar to erupt [ 23 - 25 ].

In the present study, similar to the findings of Bingül *et al*. [ 26 ], the highest rate of third molar impaction was observed in the mandible. A long and upwardly inclined ramus and relatively shorter mandibular length indicate a higher likelihood of third molar impaction in the lower jaw [ 2 ]. According to the findings of this study, unlike the study by Keerthana *et al*. [ 27 ], more than half of the patients had Class II malocclusion. This may be due to a smaller gonial angle in these patients. Additionally, genetic and ethnic factors as well as different sampling methods used in studies can contribute to variations in the results [ 27 - 28 ].

Our study results, consistent with those of Gul *et al*. [ 4 ], showed a higher prevalence of impaction in patients with a dolichofacial facial profile. In people with a dolichofacial morphology, the jaw rotates clockwise throughout growth, and the condyle grows vertically, resulting in a decreased mandibular length, which causes the tooth to erupt posteriorly. Furthermore, compared to people with fully erupted molars, those with impacted or partially erupted third molars had a deeper sigmoid notch, which may be related to face height [ 4 ]. According to the findings of some studies, anterior ramus resorption is more common in brachyfacial individuals, creating enough space for the eruption of the third molar. Considering that one of the possible causes of impaction is insufficient space in the dental arch, this could explain the lower prevalence of impaction in brachyfacial individuals [ 4 , 29 ].

Awareness of which individuals with different skeletal patterns are more susceptible to third molar impaction, as well as examining the relationship between skeletal facial dimensions and the third molar, can be clinically useful for planning and evaluating orthodontic and surgical treatments [ 30 ].

Our study showed that Class B impaction depth, Class II relationship to the anterior ramus, and mesioangular impaction angle were the most common for impacted mandibular molars. According to the literature, women between the ages of 21 and 30 are more likely to experience mesioangular impaction [ 31 - 32 ]. The third molar may be positioned between the second molar's cervical and occlusal regions due to limitations imposed by the mesioangular impaction angle [ 33 ]. Similar to the study by Sigaroudi *et al*. [ 9 ] the higher prevalence of the mesioangular pattern may be related to the initial growth position of the tooth bud, which starts with an oblique or sometimes horizontal occlusal surface. Then the growing crown adjusts to positional changes in the mandible [ 9, 34 ].

For impacted maxillary molars, the most common depth of impaction was Class C, and the most frequent impaction angle was distoangular. Unlike the lower jaw, the third molar in the upper jaw does not typically have a mesial inclination. Consequently, it is predictable that the mesioangular impaction pattern is less common in the upper jaw [ 1 ].

Our study, similar to the study by Arefi *et al*. [ 1 ], found no significant correlation between the depth of third molar impaction in both the maxilla and mandible and the vertical and anteroposterior dimensions of the face. However, the results showed a significant correlation between the level of impaction of the third molar in the mandible (distance to the anterior border of the ramus) with the vertical dimension of the face, but not with the anteroposterior dimension. Similar to the study by Bingül *et al*. [ 26 ], our results indicated a significant correlation between the angulation of third molar impaction in the mandible and both the vertical and anteroposterior dimensions of the face. These results differ from the findings of Eskandari *et al*. [ 29 ] and Demyati *et al*. [ 3 ]. This difference in the results can be related to the difference in the methods for measuring the skeletal dimensions of the face and the number of selected samples.

According to the findings of some studies, facial growth can help predict the eruption of the mandibular third molar and is associated with sufficient space in the mandible. Some have indicated that three skeletal characteristics- short mandibular length, vertical condylar growth, and posterior dental eruption- are associated with a lack of space for third molar eruption [ 26 - 27 , 35 ].

The findings of this study can be beneficial in orthodontics for assessing the potential impact of third molar impaction on dental crowding, appropriate treatment planning, and early diagnosis. Increasing knowledge about the expected changes of the third molar in the years following the completion of orthodontic treatment can provide valuable guidance for treatment planning and potentially timely referral to an oral and maxillofacial surgeon [ 36 ].

According to the findings of our study, there was no significant relationship between the angulation of maxillary third molar impaction and the anteroposterior and vertical dimensions of the face. These findings differ from the results of Bin Rubaia’an *et al*. [ 2 ]. A possible reason for this discrepancy is the differences in demographic characteristics and evaluation criteria of the samples. In the mentioned study, cone beam computed tomography (CBCT) imaging was used, whereas in our study, panoramic and cephalometric imaging were utilized.

The lack of a significant correlation between the impaction pattern of the third molar and the type of malocclusion may be because skeletal malocclusions involve discrepancies and mismatches between the jaws. A lack of space in one jaw, leading to an increased chance of third molar impaction, may be accompanied by increased space in the other jaw [ 1 ].

Differences in the prevalence of impaction patterns can also be attributed to variations in race and the studied population. Racial differences in facial growth patterns, jaw size, and tooth size-all essential factors in eruption patterns-play a role. Additionally, the criteria for classifying skeletal facial patterns differ among studies [ 8, 29 ]. 

This study had some limitations. The samples were only from patients who visited a private radiology center in Zanjan, which may limit the generalizability of the results. Additionally, the imaging methods used (panoramic and lateral cephalometry) have limitations in measurement accuracy. Lastly, an imbalanced gender distribution among the samples could have influenced the results. Given the limitations of this study, including the studied population and the use of two-dimensional imaging methods, it is suggested that future research be conducted using three-dimensional imaging (CBCT) and larger sample sizes from diverse populations. Furthermore, examining the relationship between other factors such as jaw growth, dental crowding, and evaluating the impact of orthodontic treatment on third molar impaction could enhance our understanding of this phenomenon.

## Conclusion

The results of this study showed that third molar impaction was more common in individuals with a dolichofacial growth pattern and Class II skeletal relationship. In the mandible, the most prevalent type of impaction was Class B and Class II with a mesioangular angulation, whereas in the maxilla, Class C and distoangular angulation were the most frequent. Additionally, there was a significant relationship between the angulation of mandibular third molar impaction and the vertical and anteroposterior facial dimensions. Since prevention is better than treatment, understanding the relationship between skeletal patterns and third molar impaction may help orthodontists and oral and maxillofacial surgeons identify patients at higher risk of impaction and implement early interventions.
